# Artificial Intelligence‐Based Urine Volatomic Assay for Multi‐Cancer Detection: A Multicentric Case–Control Discovery and Internal Validation Study

**DOI:** 10.1002/cnr2.70735

**Published:** 2026-11-10

**Authors:** Scott Campit, Sudharshanan Balaji, Matthew Laskowski, Thomas J. Polascik

**Affiliations:** ^1^ TOBY Inc. Austin Texas USA; ^2^ Department of Urology Wake Forest University Winston‐Salem North Carolina USA

**Keywords:** artificial intelligence, diagnostic biomarker, gas chromatography–mass spectrometry, internal validation, multi‐cancer detection, tissue of origin, urinary volatile organic compounds

## Abstract

**Background:**

Multi‐cancer detection assays may extend early‐detection efforts beyond organ‐specific screening programs and support emerging models of oncologic primary care, including age‐ and risk‐stratified multi‐cancer screening. We evaluated urine volatile organic compound profiles (volatome), an emerging biomarker source, for its association with cancer detection.

**Objective:**

To determine whether urinary volatome profiles measured by gas chromatography–mass spectrometry (GC–MS) and interpreted with artificial intelligence (AI) distinguish participants with known cancer from cancer‐free controls and support exploratory tissue‐of‐origin (TOO) and stage classification.

**Methods:**

This multicenter retrospective case–control discovery study included urine from 491 treatment‐naive participants with one of 10 cancers each and 200 cancer‐free healthy controls. Three participating sites contributed de‐identified specimens. Volatome profiles were measured by GC–MS. A proprietary ensemble tree‐based AI pipeline underwent repeated stratified fivefold internal cross‐validation. Preprocessing was fitted within each training partition. Each participant contributed one sample and was assigned only once within a given fold partition. The reported cancer‐signal operating point targeted 98% specificity. TOO analysis identified the most likely cancer type and its presumed tissue of origin.

**Results:**

The cancer‐signal classifier produced an out‐of‐fold area under the receiver operating characteristic curve (AUC) of 0.990 (95% confidence interval [CI], 0.984–0.996). At the reported 98% specificity‐optimized cutoff, sensitivity was 91.9% (451/491). The positive predictive value (PPV) was 99.1% with the cohort's 71% (491/691) cancer prevalence. Grouped TOO accuracy was 91.6% (450/491; 95% CI, 89.2%–94.1%). Among 374 cancers with a recorded stage, the early‐versus‐late detection classifier had an AUC of 0.866, and it correctly assigned 98.1% (252/257) of early‐stage cancers to the early class while recovering 25.6% (30/117) of the late‐stage cancers. Recursive removal with high feature importance values produced a small, gradual decline rather than an abrupt loss of performance across the evaluated classification tasks.

**Conclusions:**

Urinary VOC profiles contained a composite signal that discriminated known cancer cases from cancer‐free controls under internal cross‐validation with signals that were able to identify TOO with reasonable certainty. The feature‐removal analysis was consistent with distributed predictive information rather than reliance on one dominant VOC or a small feature panel. Prospective blinded validation in the intended‐use population remains necessary to establish population‐screening accuracy, comparative superiority, or clinical utility.

**Abbreviations**
AI: artificial intelligence
AUC: area under the ROC curve
FIGO: The International Federation of Gynecology and Obstetrics
FIT: fecal immunochemical test
GC–MS: gas chromatography–mass spectrometry
HPLC: high‐performance liquid chromatography
IRB: Institutional Review Board
MCED: multi‐cancer early detection
OOF: out‐of‐fold
ROC: receiver operating characteristic
TOO: tissue‐of‐origin
VOC: volatile organic compound

## 1 Introduction

Earlier cancer diagnosis can increase the opportunity for curative treatment in select cancers while reducing disease‐ and treatment‐related morbidity, along with mortality [1, 2, 3]. Current population screening remains organ‐specific and is recommended only for a subset of cancers and risk groups. Several lethal cancers, including pancreatic and ovarian cancer, lack established population‐screening programs [4]. This gap has accelerated the development of multi‐cancer detection assays. Their clinical value, however, depends on performance in the intended‐use population and on the diagnostic pathway that follows a positive result.

Most multi‐cancer detection approaches under clinical evaluation use blood‐based analytes, including circulating cell‐free DNA [5, 6, 7, 8, 9, 10]. Sensitivity varies by cancer type and stage. Several assays also predict the likely tissue of origin to guide diagnostic evaluation [6, 7, 9, 10]. Results from enriched case–control cohorts cannot be transferred directly to population screening because disease prevalence, disease spectrum, comorbidity, verification procedures, and downstream harms differ.

Volatile organic compounds (VOCs) in urine and breath have been studied as cancer‐associated biomarkers [11, 12, 13, 14, 15, 16, 17, 18, 19]. Collectively, compounds constitute the volatome, the complete set of volatile metabolites a biological sample emits. Urinary volatome profiles integrate tumor metabolism with host response, microbiome activity, diet, medications, renal handling, environmental exposure, and pre‐analytical conditions. Most published urinary studies address one cancer at a time or tend to be centered around a single biomarker or a specific pathway. Multi‐cancer signal detection and tissue‐of‐origin (TOO) classification using urinary VOCs remain discovery‐stage applications.

This study evaluated whether a urine volatomic profile measured by gas chromatography–mass spectrometry (GC–MS) and interpreted with artificial intelligence (AI) could distinguish participants with known cancer from cancer‐free controls. Secondary analyses examined grouped and individual tissue of origin and cancer stage. The work represents model development and internal validation within a multicentric retrospective case–control cohort.

## 2 Materials and Methods

### 2.1 Study Cohort

Three participating sites contributed de‐identified urine and clinical data. The cohort included participants with breast, prostate, lung, colorectal, bladder, kidney, pancreatic, stomach, ovarian, or cervical cancer and cancer‐free controls. Cancer participants had a site‐recorded diagnosis and provided urine before cancer‐directed treatment. No participant was receiving systemic therapy, radiotherapy, treatment directed to the primary tumor, or treatment directed to metastatic sites before collection. Cancer‐free controls were described as self‐reported healthy and were not known to have any of the 10 study cancers. Their evaluation reflected routine annual primary care and age‐appropriate usual care. The protocol did not require imaging, endoscopy, or laboratory testing to exclude occult malignancy. Available cancer type, stage, and grade were provided by the participating sites, and tumor histopathology was used to confirm diagnosis. The stage used American Joint Committee on Cancer (AJCC) eighth‐edition categories [20]. Supplied International Federation of Gynecology and Obstetrics categories were mapped to the corresponding stage labels in the study dataset. Stage analyses included only cases with a recorded stage.

### 2.2 Sample Collection and Handling

Midstream clean‐catch urine was collected in a sterile specimen cup, immediately chilled at 4°C, and subsequently frozen at 80°C. Frozen aliquots were shipped on dry ice to the central testing laboratory before analysis. Data on collection time, collection‐to‐cooling and collection‐to‐freezing intervals, fasting status, prior freeze–thaw history, medication use, and other clinical covariates were unavailable. All samples were analyzed from frozen storage and randomized across analytical runs; no samples were analyzed fresh. After thawing and centrifugation, urine aliquots were diluted with high‐performance liquid chromatography (HPLC)‐grade water and normalized to an internal standard. VOCs were extracted using a commercial stir bar sorptive extraction device (Twister, GERSTEL GmbH, Linthicum, MD, USA), followed by thermal desorption and GC–MS analysis. Quality control procedures were applied consistently across all samples. Processed GC–MS runs were required to meet predefined acceptance criteria for an internal reference standard, including retention time and scan number windows. Samples that did not satisfy these criteria were flagged for review and rerun as needed before score generation. Laboratory personnel remained blinded to case–control status during sample preparation, GC–MS acquisition, and initial data processing. No technical replicates, duplicate aliquots, or triplicate measurements were included. Each participant contributed one analyzed sample.

### 2.3 GC–MS Data Capture and Processing

After thawing and centrifugation, 1 mL of urine was diluted with HPLC‐grade water and prepared with an internal standard. VOCs were extracted using a Twister stir‐bar sorptive extraction device (GERSTEL), thermally desorbed, and analyzed on an Agilent 8890 gas chromatograph (Agilent Technologies, Santa Clara, CA, USA) coupled to a 5977B mass‐selective detector with GERSTEL TDU/CIS automation. Spectra were converted to mzML using an in‐house native reader, then deconvolved in the style of AMDIS/ADAP‐GC with baseline removal, Savitzky–Golay smoothing, per‐ion peak detection, and greedy grouping of co‐apexing ions into model peak components with custom parameters. Components were matched across samples by anchor‐based retention‐time warping followed by weighted‐cosine clustering at a specified cosine threshold. A retention time tolerance of ±5 scans was used after anchor‐based retention time correction. The internal standard was identified within ±0.17 min of its expected retention time. Tentative compound identities were assigned by comparison with the National Institute of Standards and Technology (NIST26) and Automated Mass Spectral Deconvolution and Identification System mass‐spectral libraries using NIST MS Search. The search retained the top hits per query and scored each as the geometric mean of forward and reverse match factors. Retained compound‐level signals were represented as Chemical Abstracts Service‐indexed features, with abundances as summed deconvolved component areas. Feature extraction, scaling, and any batch‐effect handling were performed within a standardized preprocessing pipeline consistent with the locked model. Probabilistic quotient normalization (PQN) was used to reduce dilution‐related variation.

### 2.4 Metabolomics Data Processing

Compound‐level features were screened against the Keller contaminant list [21], VOCBinBase [22], and the NORMAN Suspect List Exchange [23]. Features without a resolved common chemical name and a biological annotation in ChEBI [24] or LIPID MAPS [25] were excluded. Within each training partition, PQN [26], median imputation, robust scaling by the median and interquartile range, and class‐weight calculation were fitted with training data and then applied to the held‐out partition. Compound values that were not detected were set to 0. No feature values were missing in the data supplied to the imputation step. Neither missingness indicators nor principal component analysis were used. The final feature‐processing rules were fixed before the reported internal‐validation run. Because pipeline development and internal validation used the same overall cohort, these procedures do not constitute independent external feature validation.

### 2.5 Statistical Analysis

Exploratory univariable comparisons used PQN‐normalized feature abundances. The log_2_ ratio of the cancer and control medians summarized the effect size. Two‐sided Mann–Whitney *U* tests compared groups. The Benjamini–Hochberg procedure controlled the false discovery rate within each comparison. A *q* value below 0.05 identified evidence of differential abundance. These within‐cohort associations were used for description and biological interpretation. They were not treated as independent biomarker validation.

Discrimination was summarized using the area under the receiver operating characteristic (ROC) curve (AUC). At the reported high‐specificity operating point, confusion‐matrix counts, sensitivity, specificity, precision, and *F*1‐score were calculated as applicable. Grouped TOO performance was summarized using accuracy, macro and weighted *F*1‐scores, and class‐specific sensitivity. Age‐restricted, age‐matched, age‐plus‐sex, and participating‐site classifiers were evaluated on the same fold assignments as bias probes. A multi‐shuffle label‐permutation test was used to assess discrimination above chance. Labels were independently permuted 200 times. Each permutation was rescored with the cross‐validation procedure, and a one‐sided, add‐one‐corrected empirical *p* value was computed against the null AUC distribution.

### 2.6 Model Training

Multiple classifiers were trained using a proprietary ensemble tree‐based AI framework that returned discrete probabilities. The specific framework is not disclosed to protect intellectual property associated with the assay; this restriction limits independent reproduction of the computational pipeline. Internal performance was estimated by repeated stratified fivefold cross‐validation [27]. For each classification task, five repeats were used for cross‐validation. A random seed value of 42 was set for reproducibility. New fold assignments were generated for each repeat. No technical replicate or duplicate sample could cross partitions. Stratification used overall cancer‐versus‐control status for cancer detection, cancer tissue type for TOO classification, early/late stage labels, and individual cancer stage labels. An unweighted arithmetic mean of the participant's out‐of‐fold (OOF) probabilities was used to aggregate predictions to one pooled probability. Each sample entered the fold assignment once. De‐identified participant identifiers were used to prevent a participant from appearing in both the training and held‐out partitions of the same fold. Preprocessing was estimated independently in each training partition and applied to its held‐out partition. The final pipeline was fixed before the reported internal validation run. No independent holdout or external validation cohort was used.

The cancer detection threshold was selected to achieve a specificity of 98%, reflecting the intended use of the assay as a first‐line, noninvasive triage test within a population where the target cancers have low individual prevalence. The candidate set of operating points is from the observed pooled OOF probabilities in 0.005 steps above the minimum threshold of 0.98, and the final operating point is selected from the nearest attainable empirical value from the argmax of the sensitivity. The classification threshold is selected from the pooled OOF predictions.

### 2.7 Model Validation

Classifier 1 distinguished cancer from control. Classifier 2a assigned one of four grouped tissues of origin: genitourinary (bladder, kidney, and prostate), breast and gynecologic (breast, cervical, and ovarian), gastrointestinal (colorectal, pancreatic, and stomach), or pulmonary (lung). Each group maps to one referral specialty (urology, breast/gynecologic oncology, gastroenterology, or pulmonology), rather than by anatomical grouping. This grouping is constrained by the 10 tumor types available in this cohort and is not proposed as a general organ‐system taxonomy.

Classifier 2b correctly detected cancers from Classifier 1 and assigned each sample one of 10 individual tissues of origin (bladder, kidney, prostate, breast, cervical, ovarian, pancreas, lung, stomach, and colorectal). The model returns a probability across all 10 tissues for each sample, which is ranked from most to least likely. Performance was evaluated using Top‐1 and Top‐2 accuracy, which are the fraction of samples whose true tissue was the highest ranked prediction and the fraction whose true tissue appeared among the two highest ranked predictions.

Classifier 3a distinguished early (Stages I/II) from late (Stages III/IV) using recorded AJCC, FIGO, and TNM stages when available, providing 374 early and late labels. Classifier 3b assigned one of four stage labels with recorded AJCC and FIGO stages only, yielding 355 stage labels out of 491 cancers. TOO and stage classifiers were evaluated independently among cancer cases rather than only among samples classified as cancer‐signal positive.

Cancer detection discrimination was summarized using the AUC. The reported 98% specificity operating point was evaluated using confusion matrix counts, sensitivity, specificity, precision, and *F*1 score. Grouped TOO performance used overall accuracy, macro and weighted *F*1‐scores, and class‐specific sensitivity. Participating‐site and age‐plus‐sex classifiers served as descriptive bias probes on the same fold assignments. Permutation tests were run, as described in the Section 2.5. All reported estimates represent internal validation.

### 2.8 Model Robustness Analyses

Model robustness and the impact of any single VOC were assessed by removing VOCs sequentially and evaluating their performance on cancer detection, TOO classification, organ‐system‐of‐origin, and cancer stage classification. VOCs were removed one at a time without replacement. During each VOC removal, the model was refitted with the existing pipeline, and feature importances were recalculated. The VOC with the highest feature importance value was selected and removed until there were no VOCs in the dataset. During each VOC removal, the classifier was rescored. All preprocessing and model fitting were confined to training partitions, and predictions were OOF. Statistical significance at each VOC removal step was assessed against a max‐statistic null constructed from *N* = 200 anchors. 95% confidence intervals (CIs) were obtained from a 2000‐resample percentile bootstrap (except for the macro recall values). No sample was duplicated across folds, and no technical replication entered the analysis.

## 3 Results

### 3.1 Study Cohort

The study included 691 participants: 491 with one of 10 cancers and 200 cancer‐free controls (Figure 1; Table 1). Three participants (0.61%) had associated clinical text that described additional diagnoses of more than 1 cancer from the primary site. For those cases, the primary cancer site was used as the label. Cancer samples were included based on biobank‐annotated primary cancer diagnoses corresponding to one of the 10 tumor types studied. Information on additional primary or synchronous malignancies was not systematically captured by the source biobanks, and patients were therefore not excluded on this basis. One participating site contributed all 200 controls and 59 cancer patients. The other two sites contributed cancer cases only. Cancer and control groups differed in age, sex, and race. Cancer type and stage also varied across participating sites (Table S1). These cohort features remain relevant because participant‐level cross‐validation does not by itself separate disease‐related signals from site‐associated or pre‐analytical signals. Because information regarding additional primary or synchronous malignancies was not systematically captured, we cannot formally confirm the absence of multiple primary or synchronous cancers in all patients.

**FIGURE 1:**
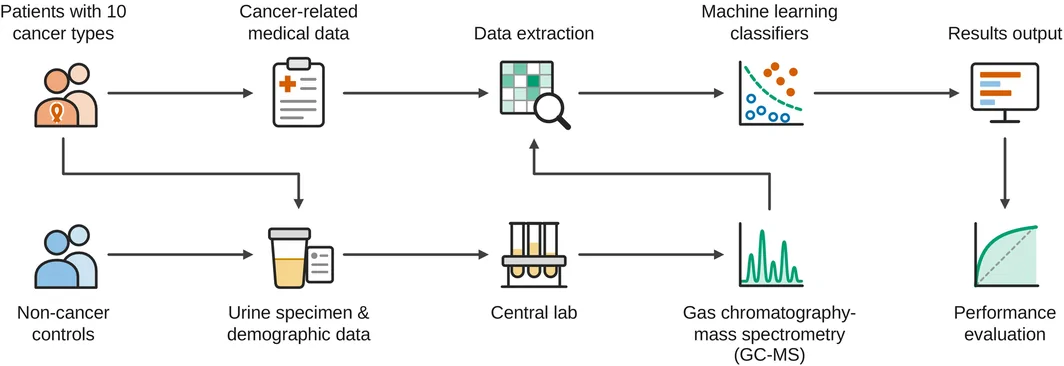
Study design. The schematic was created using Claude Design (Anthropic).

**TABLE 1:** Demographic and clinical characteristics.

Characteristic	Cancer (*n* = 491)	Non‐cancer controls (*n* = 200)
Age, years ± SD (range)	59.0 ± 9.7 (29–88)	41.0 ± 12.5 (18–71)
Sex, *n* (%)		
Male	194 (39.5%)	100 (50.0%)
Female	297 (60.5%)	100 (50.0%)
Race, *n* (%)		
Caucasian	462 (94.1%)	60 (30.0%)
African American	21 (4.3%)	70 (35.0%)
Hispanic/Latino	0 (0.0%)	69 (34.5%)
Pacific Islander	2 (0.4%)	0 (0.0%)
Asian	0 (0.0%)	1 (0.5%)
Unknown	6 (1.2%)	0 (0.0%)
Cancer type, *n* (%)		
Breast	84 (17.1%)	NA
Prostate	24 (4.9%)	NA
Lung	25 (5.1%)	NA
Colorectal	50 (10.2%)	NA
Bladder	87 (17.7%)	NA
Kidney	49 (10.0%)	NA
Pancreas	25 (5.1%)	NA
Stomach	50 (10.2%)	NA
Ovarian	65 (13.2%)	NA
Cervical	32 (6.5%)	NA
Tumor grade, *n* (%)		
1	17 (3.5%)	NA
2	51 (10.4%)	NA
3	16 (3.3%)	NA
4	0 (0.0%)	NA
Unknown	407 (82.9%)	NA
AJCC stage group, *n* (%)		
I	174 (35.4%)	NA
II	83 (16.9%)	NA
III	63 (12.8%)	NA
IV	35 (7.1%)	NA
Unknown	136 (27.7%)	NA
Stage resolution, *n* (%)		
Exact AJCC group (I–IV)	355 (72.3%)	NA
Bounded by TNM to III/IV	19 (3.9%)	NA
Bounded by TNM to 0/I/II	0 (0.0%)	NA
Unresolved	117 (23.8%)	NA
Gleason score, *n* (%)		
6	12 (2.4%)	NA
7	11 (2.2%)	NA
8	1 (0.2%)	NA
9–10	0 (0.0%)	NA
Not applicable (non‐prostate)	467 (95.1%)	NA
Unknown	0 (0.0%)	NA
Risk group, *n* (%)		
Low‐risk	12 (2.4%)	NA
High‐risk	12 (2.4%)	NA
Not applicable (non‐prostate)	467 (95.1%)	NA
Unknown	0 (0.0%)	NA

After PQN, the overall feature‐intensity distributions were broadly similar in shape between cancer cases and controls (Figure 2A). Selected VOCs differed between groups (Figure 2B). These exploratory distributions may reflect cancer biology, participant characteristics, environmental exposure, or pre‐analytical variation. Literature annotations for disclosed features appear in Table S2 [28, 29, 30, 31, 32, 33, 34, 35, 36, 37, 38].

**FIGURE 2:**
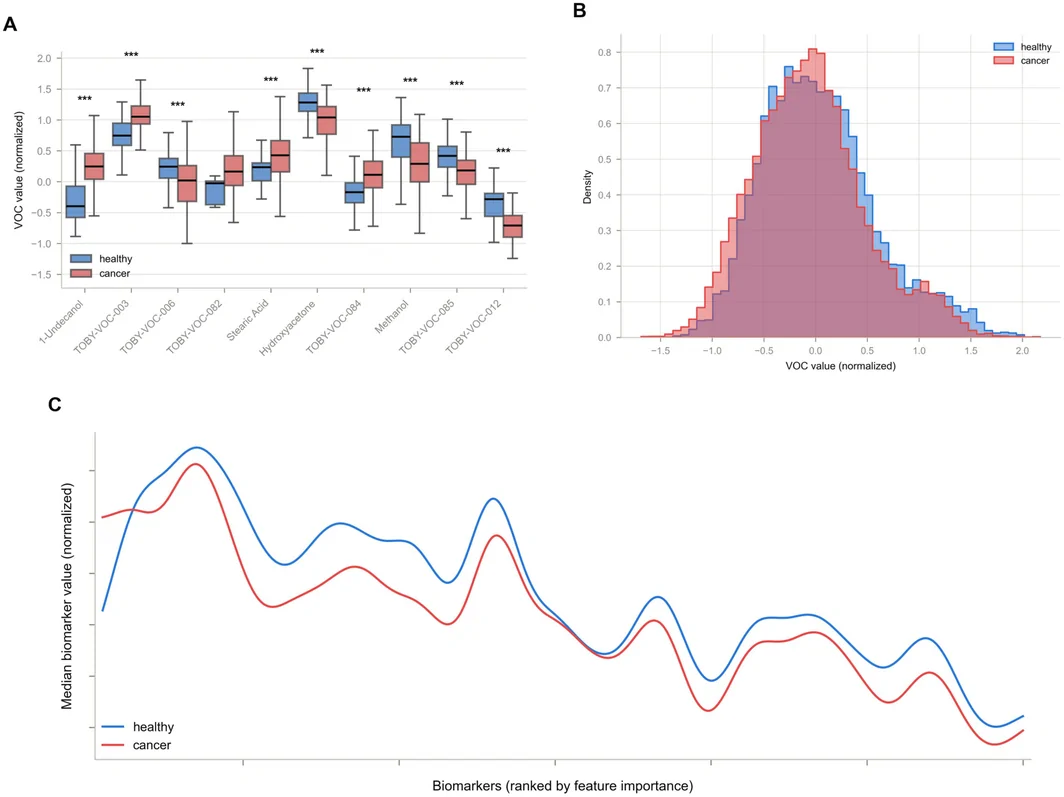
Urinary VOC abundance distributions in cancer and control samples. (A) Distributions of selected features. Group differences were assessed using two‐sided Mann–Whitney U tests with Benjamini–Hochberg correction. *q < 0.05; **q < 0.01; ***q < 0.001. (B) Distribution of log_10_‐transformed PQN‐normalized feature values between cancer and health patients. (C) log_10_‐transformed PQN‐normalized VOC profiles, in descending order by median values. Red denotes cancer samples and blue denotes controls. These plots are exploratory.

### 3.2 Cancer Signal Detection

The cancer‐signal classifier yielded an OOF AUC of 0.990 (95% CI, 0.984–0.996; Figure 3A). At the reported operating point, specificity was 98% (196/200) and sensitivity was 91.9% (451/491; 95% CI, 89.1%–94.1%; Figure 3B) with 99.1% PPV. Results by demographic group and participating site appear in Table 2. In the only site that contributed both cancer cases and controls, the AUC was 0.936, and sensitivity was 49.2% (29/59; 95% CI, 35.9%–62.5%) at 98% specificity. The remaining site‐specific estimates describe sensitivity only because those sites did not contribute controls.

**FIGURE 3:**
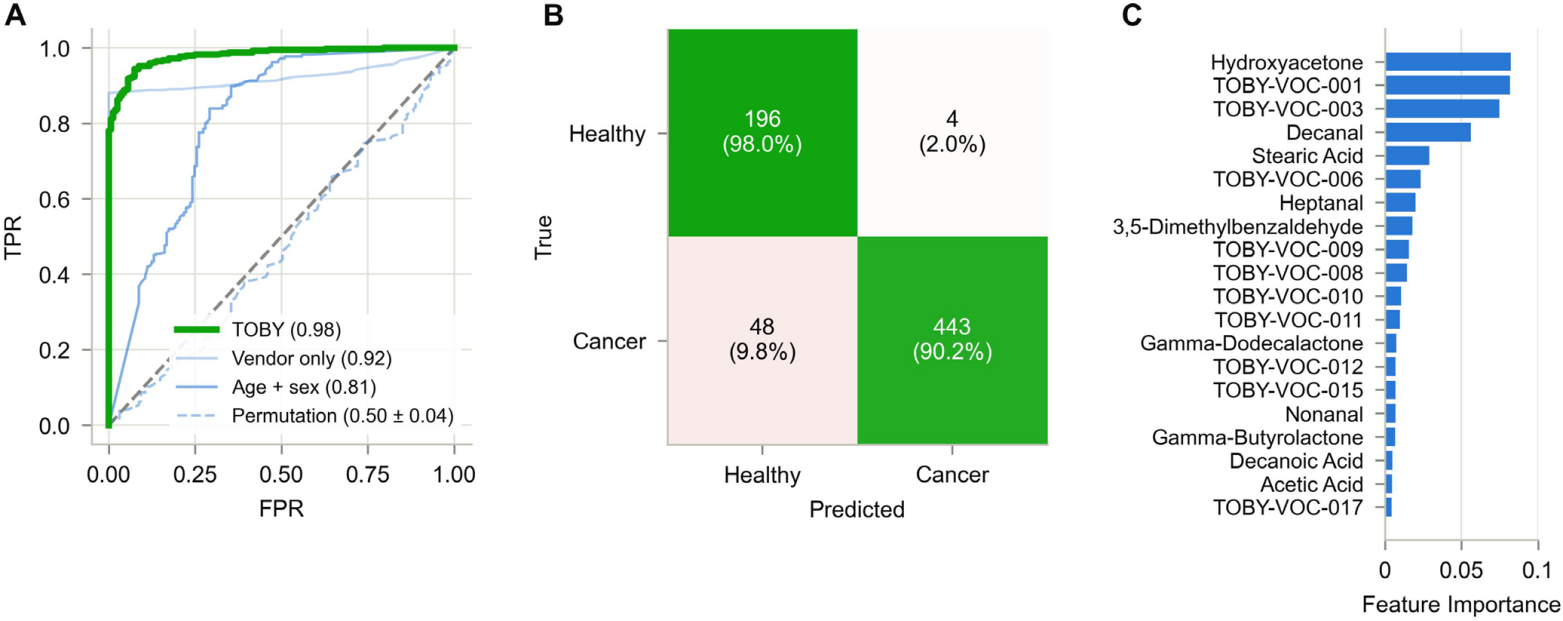
Cancer‐signal classifier performance and feature importance from out‐of‐fold predictions. (A) Receiver operating characteristic curve. (B) Confusion matrix at the reported high‐specificity operating point. (C) Twenty highest‐ranked features.

**TABLE 2:** Cancer‐signal detection performance by demographic group and participating site.

Attribute	Level	*N*	*N* (cancer)	*N* (control)	AUC	Sensitivity	Specificity
Overall	All	691	491	200	0.990	92% (451/491)	98% (196/200)
Sex	Male	294	194	100	0.989	89% (172/194)	98% (98/100)
	Female	397	297	100	0.991	94% (279/297)	98% (98/100)
Age (years)	> 45	525	451	74	0.987	91% (412/451)	96% (71/74)
	≤ 45	166	40	126	0.998	98% (39/40)	99% (125/126)
Race	Caucasian	522	462	60	0.988	91% (422/462)	98% (59/60)
	Non‐Caucasian	163	23	140	0.999	100% (23/23)	98% (137/140)
Participating site	BioIVT	259	59	200	0.936	49% (29/59)	98% (196/200)
	EVMS	101	101	0	NA	100% (101/101)	NA
	iSpecimen	331	331	0	NA	97% (321/331)	NA

*Note:* Six participants had missing race and were omitted from the race‐stratified AUC, sensitivity, and specificity calculations.

Abbreviation: EVMS, Eastern Virginia Medical School.

Using the same cancer‐versus‐control classifier and operating point, sensitivity was 98% (170/174) for Stage I, 90% (75/83) for Stage II, 89% (56/63) for Stage III, and 94% (33/35) for Stage IV disease (Table 3). These are stage‐stratified cancer‐detection sensitivities. They do not measure the accuracy of a model that predicts stage and remain influenced by the cancer type and participating site composition of each stage group.

**TABLE 3:** Cancer signal detection sensitivity results with 98% specificity.

Cancer type	All	Stage I	Stage II	Stage III	Stage IV	Unknown	Specificity
All (micro)	92% (451/491)	98% (170/174)	90% (75/83)	89% (56/63)	94% (33/35)	86% (117/136)	98% (196/200)
Breast	94% (79/84)	98% (49/50)	88% (14/16)	89% (16/18)	NA	NA	
Prostate	100% (24/24)	NA	NA	NA	NA	100% (24/24)	
Lung	100% (25/25)	100% (25/25)	NA	NA	NA	NA	
Colorectal	98% (49/50)	97% (29/30)	100% (20/20)	NA	NA	NA	
Bladder	67% (58/87)	NA	44% (4/9)	50% (5/10)	67% (2/3)	72% (47/65)	
Kidney	98% (48/49)	NA	NA	NA	100% (2/2)	98% (46/47)	
Pancreas	100% (25/25)	100% (19/19)	100% (6/6)	NA	NA	NA	
Stomach	96% (48/50)	94% (17/18)	97% (31/32)	NA	NA	NA	
Ovarian	98% (64/65)	NA	NA	100% (35/35)	97% (29/30)	NA	
Cervical	97% (31/32)	97% (31/32)	NA	NA	NA	NA	

*Note:* All (micro) pools every cancer; NA marks a cancer type with no cases at that stage.

The 20 highest‐ranked features appear in Figure 3C, with Shapley additive explanation summaries in Figure S1. Several disclosed compounds have been reported in urinary or other cancer‐associated metabolomic studies [28, 29, 30, 31, 32, 33, 34, 35, 36, 37, 38] (Table S2). The strength, biological matrix, direction, and disease specificity of that evidence vary. Literature concordance supports plausibility but does not establish that a feature is cancer‐specific, tumor‐derived, or independently validated.

### 3.3 TOO Classification

Among 491 cancer cases, the grouped TOO classifier had a macro one‐versus‐rest AUC of 0.975 (Figure 4A) and an accuracy of 91.6% (450/491; 95% CI, 89.2%–94.1%; macro‐*F*1, 0.865; weighted‐*F*1, 0.914; Figure 4B). Sensitivity ranged from 96% (174/181) for the reproductive group to 52% (13/25) for the pulmonary group (Table 4). This analysis evaluated four clinically broad tissue groupings and should not be interpreted as exact anatomic localization.

**FIGURE 4:**
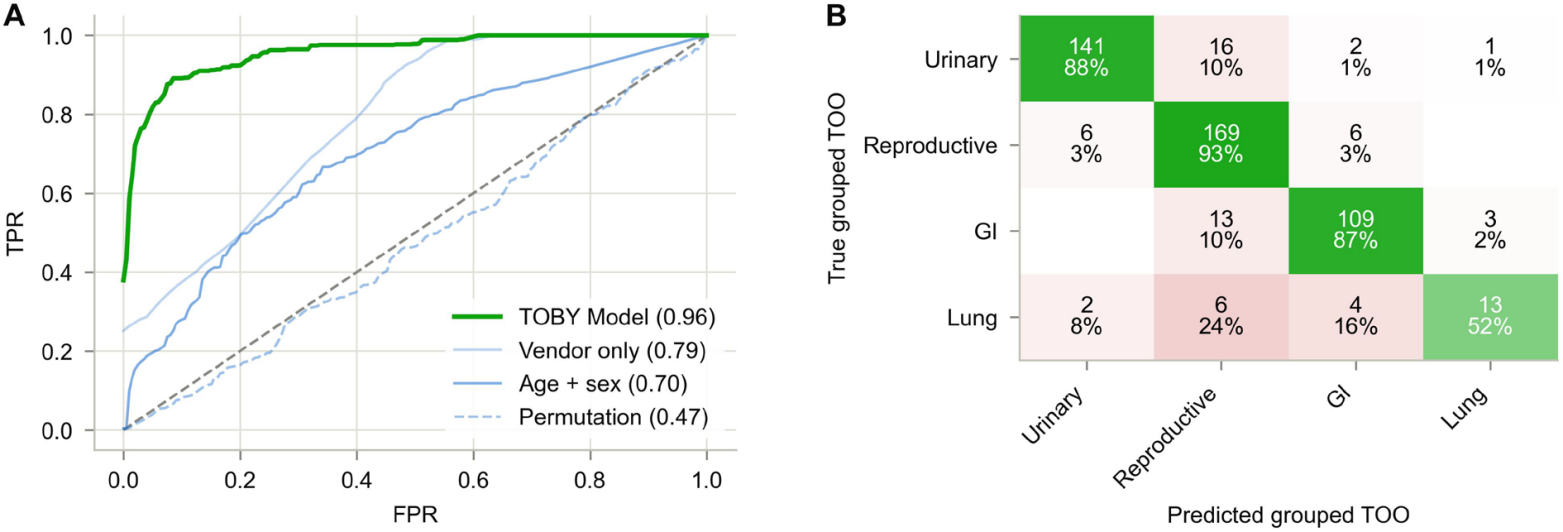
Grouped tissue‐of‐origin classifier performance among cancer cases. (A) One‐versus‐rest receiver operating characteristic curves. (B) Top‐1 grouped tissue‐of‐origin confusion matrix.

**TABLE 4:** Grouped tissue‐of‐origin sensitivity by tissue grouping and cancer stage.

Grouped tissue of origin	All	Stage I	Stage II	Stage III	Stage IV	Unknown
All (micro)	92% (450/491)	87% (152/174)	92% (76/83)	94% (59/63)	94% (33/35)	96% (130/136)
Urinary	95% (152/160)	NA	100% (9/9)	80% (8/10)	100% (5/5)	96% (130/136)
Reproductive	96% (174/181)	96% (79/82)	100% (16/16)	96% (51/53)	93% (28/30)	NA
GI	89% (111/125)	90% (60/67)	88% (51/58)	NA	NA	NA
Lung	52% (13/25)	52% (13/25)	NA	NA	NA	NA

*Note:* NA marks a group with no cases at that stage.

Individual TOO classification across the 10 cancers was more difficult than grouped TOO classification (Top‐1 accuracy = 77.0%, 378/491; Top‐2 = 90.4%, 444/491). Of the 113 Top‐1 misclassification errors, 78 (69%) of the misclassifications are assigned to the same organ system. Exploratory Top‐1 and Top‐2 results appear in Figure S2. The individual TOO analysis was performed independently among cancer cases.

### 3.4 Early/Late Cancer Stage and Stage‐Group Classification

Among 374 cancer cases with a resolvable early and late stage, the early‐versus‐late classifier yielded an AUC of 0.866 (Figure 5A). With late stage treated as the positive class at the reported operating point, sensitivity was 26% (30/117), and specificity was 98% (252/257). Overall accuracy was 75% (282/374), which largely reflected correct classification of the larger early‐stage group. Stage‐specific classifiers were fitted to predict individual cancer stages among the 355 cases with an exact stage group. The four‐class stage model yielded a macro one‐versus‐rest AUC of 0.786. Sensitivity was 80% (139/174) for Stage I, 22% (18/83) for Stage II, 43% (27/63) for Stage III, and 17% (6/35) for Stage IV disease (Figure 6; Table 5). These results support an exploratory rather than clinically actionable interpretation of stage assignment.

**FIGURE 5:**
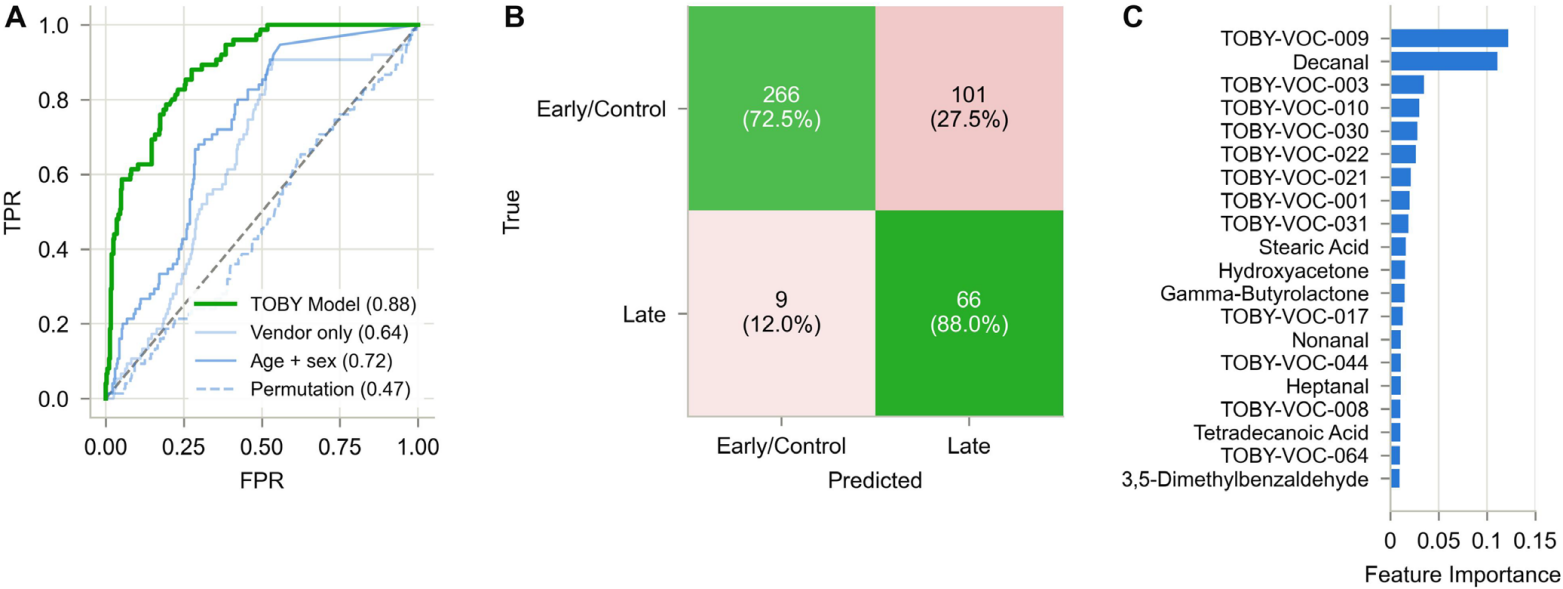
Exploratory early‐versus‐late‐stage classification among cancer cases with recorded stage. (A) Receiver operating characteristic curve for the early (I/II) versus late (III/IV) stage. (B) Confusion matrix. The late stage is the most positive class for reported sensitivity. (C) Twenty highest‐ranked features.

**FIGURE 6:**
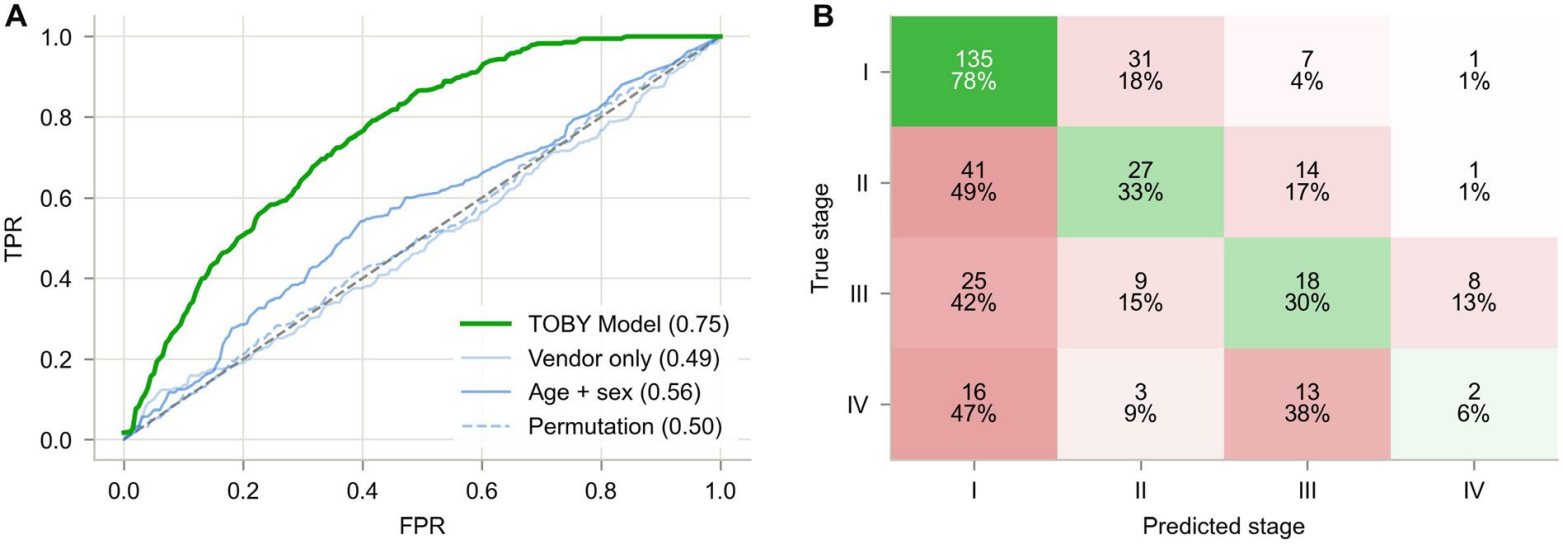
Exploratory four‐class AJCC stage classification among cancers with known stage. (A) One‐versus‐rest receiver operating characteristic curves. (B) Confusion matrix for Stages I–IV.

**TABLE 5:** Cancer stage classification sensitivity and specificity.

Classification	AUC	Stage‐assignment recall	Specificity
Late stages (III–IV) vs. early stages (I–II)	0.866	26% (30/117)	98% (252/257)
Stage I vs. Stages II–IV	0.791	80% (139/174)	51% (93/181)
Stage II vs. Stages I, III, and IV	0.750	22% (18/83)	88% (239/272)
Stage III vs. Stages I, II, and IV	0.826	43% (27/63)	90% (264/292)
Stage IV vs. Stages I–III	0.778	17% (6/35)	95% (304/320)

### 3.5 Demographic and Participating‐Site Analyses

Cancer and control participants differed in age (median_case_ = 59, range_case_ = 29–88 years old; median_control_ = 40, range_control_ = 18–71 years old), sex, and race (Table 1; Figure 7A,B). In descriptive bias probes, the age‐plus‐sex classifier yielded an AUC of 0.814, the volatomic classifier an AUC of 0.990, and the combined classifier an AUC of 0.992 (Figure 7C). The analysis restricted to the site that contributed both cases and controls yielded an AUC of 0.983 against 0.892 for its matched age‐plus‐sex model.

**FIGURE 7:**
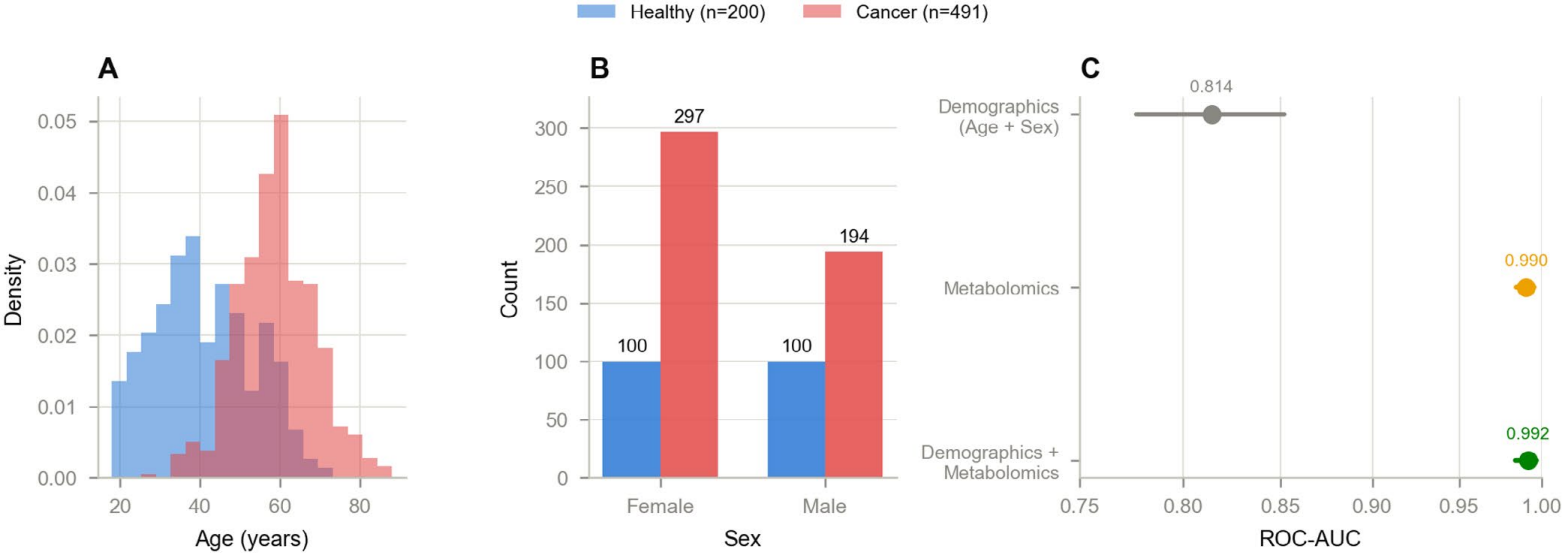
Demographic and participating‐site bias probes for cancer‐signal classification. (A) Age distribution by cancer status. (B) Sex distribution by cancer status. (C) Out‐of‐fold AUCs for age‐plus‐sex, volatilomic, and combined models. These comparisons do not exclude residual participating‐site or unmeasured confounding.

To address the differences in age range, predictions were rescored with an age‐restricted and age‐matched subset. For the age‐restricted subset (29–71 years old, *N* = 607), the resulting AUC was 0.991, compared to age alone with an AUC of 0.818. A greedy 1:1 caliper matching on age (±3 years; *N* = 222) gave an AUC of 0.994, while age alone had an AUC of 0.519. These analyses show that age and sex alone did not reproduce the full volatomic AUC. They do not exclude residual confounding by participating site, cancer type, unmeasured clinical variables, or pre‐analytical handling.

### 3.6 Sequential Feature‐Removal Robustness Analysis

Sequential removal of highly ranked features produced a gradual decline in performance rather than an abrupt collapse in any of the four evaluated tasks (Figure 8). Cancer‐signal AUC (Figure 8A) declined smoothly from 0.990, not reaching random chance (AUC = 0.50) until removing 300 of 335 biomarkers. Grouped TOO accuracy held above 0.80 for the first 200 removals and then collapsed (Figure 8B). Early‐versus‐late AUCs degraded the fastest, reaching random chance with more than 40% of its biomarkers still present (Figure 8C). This pattern supports a distributed multivariable signal. It does not establish comparative superiority to conventional cancer‐screening tests because those reference values arise from different populations and study designs.

**FIGURE 8:**
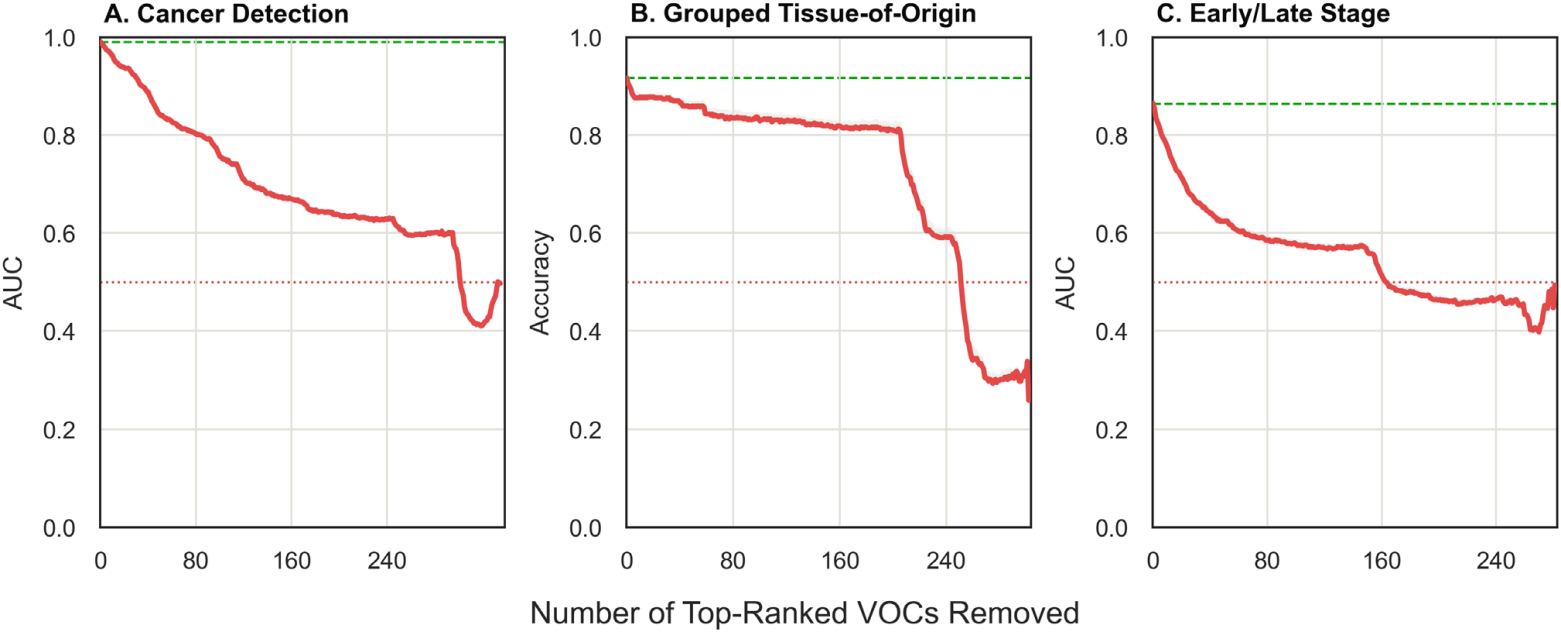
Recursive feature‐removal robustness analysis. Performance is shown after cumulative removal of VOCs with high feature importance values (descending) for (A) cancer‐signal detection, (B) grouped tissue‐of‐origin classification, and (C) early‐versus‐late stage classification. VOCs were removed sequentially, one at a time, without replacement. Models were refitted, and feature importances were calculated before dropping the next VOC. The green reference lines on the *y*‐axis represent the baseline performance of the fully trained model. Red reference lines at *y* = 0.50 indicate the value for random chance.

## 4 Discussion

### 4.1 Interpretation of the Internally Validated Findings

This multicenter retrospective case–control study identified a urinary VOC pattern that distinguished known cancer cases from cancer‐free controls under repeated internal cross‐validation. The principal result was the cancer‐signal classifier. Grouped and individual TOO classification and stage assignment were secondary exploratory tasks. The study shows that the analytical profile contains a reproducible within‐cohort signal. It does not establish performance in an asymptomatic screening population.

Direct numerical comparison with mammography, low‐dose CT, fecal immunochemical testing (FIT), or other multi‐cancer early detection (MCED) assays is not appropriate because those studies used different populations, prevalence, endpoints, reference standards, and validation designs [39, 40, 41, 42, 43]. This case–control study also cannot estimate positive or negative predictive value in an intended‐use population. The relevant next comparison is a prespecified, independently validated pathway study in which TOBY is evaluated alongside usual care in the target population.

The cancer‐signal classifier detected a high proportion of Stage I cases in this cohort. This finding represents stage‐stratified cancer‐detection sensitivity, not validated early‐cancer screening sensitivity. Stage distribution differed across cancer types and participating sites, and the controls did not constitute a prospectively enrolled screening population. The separate stage‐prediction models performed less well. Late‐stage sensitivity was 26% at the reported operating point, and Stage IV sensitivity in the four‐class model was 17%. Stage prediction therefore remains exploratory.

### 4.2 Demographic, Site, and Pre‐Analytical Considerations

Age, sex, race, cancer type, stage, and participating site were heterogeneous, spanning a broad range of the demographic and clinical variation encountered in practice. Cancer prevalence in this cohort was set by the case–control sampling design and does not reflect that of a screening population.

Surprisingly, the volatomics model outperformed the age‐plus‐sex model, and adding age and sex produced only a small reported AUC increase, although VOC profiles vary with sex and age [44, 45]. In addition, other experiments controlling for age ranges and age matching outperformed their age‐based control models. This suggests that the model used information beyond those two recorded variables. It does not establish that the signal was cancer‐specific because smoking, diet, medication use, renal function, infection, comorbidity, hospitalization, collection time, and handling variables could be potential effect modifiers or confounders.

### 4.3 Biological Plausibility and Feature Interpretation

Cancer can alter central‐carbon, redox, and lipid metabolism [46, 47, 48, 49, 50, 51, 52], creating a biologically plausible basis for metabolomic investigation. Urinary VOC abundance, however, reflects integrated tumor, host, microbiome, dietary, environmental, medication, renal, and pre‐analytical influences. Consequently, biological plausibility should be used to generate hypotheses rather than to infer that a discriminatory feature is tumor‐derived or cancer‐specific.

The feature‐removal analysis addresses a distinct validation question. Performance declined gradually as highly ranked VOCs were removed across cancer detection, Top‐1 tissue of origin, grouped tissue of origin, and early‐versus‐late‐stage classification (Figure 8). The pattern is consistent with a composite volatomic signal rather than dependence on one dominant biomarker or a small, fixed panel. Because ranking and model refitting occurred within this study cohort, the experiment remains an internal robustness analysis. It does not prove independence from all individual features or comparative superiority over established screening tests. Published AUC values for organ‐specific tests may be shown as contextual reference ranges only. Differences in populations, specimen matrices, endpoints, and validation designs preclude head‐to‐head inference.

The biological interpretation remains provisional. Urinary VOC abundance reflects tumor, host, microbiome, diet, medication, renal, environmental, and handling effects. Several influential features showed directions that differed from reports in other biological matrices. Such discordance may arise from matrix‐specific partitioning, renal handling, exposure, or analytical differences. A recent systematic review of urinary and cellular GC–MS volatomics also identified substantial methodological heterogeneity across urological cancer studies [53]. Replication should use prespecified identities, authentic standards where feasible, and specimens collected independently from model development.

### 4.4 TOO Classification

Grouped TOO accuracy was 91.6%, although performance varied by class and was lowest for lung cancer. Other MCED studies have used TOO prediction to direct diagnostic evaluation [9, 43]. The rationale for grouped TOO analysis is twofold. First, each group maps to one referral specialty (urology, breast/gynecologic oncology, gastroenterology, or pulmonology). This grouping is constrained by the 10 tumor types available in this cohort and is not proposed as a general organ‐system taxonomy. Second, compared to blood‐based MCED tests where tissue‐specific epigenetic marks provide a direct signal, volatomic profiles encode site information indirectly through metabolic and compartmental differences. From individual TOO results, we show that most errors were assigned to related tissue systems, suggesting that metabolic signatures in urine are diffused across adjacent tissues. Cross‐study numerical comparisons remain descriptive because cohort composition, disease prevalence, reference standards, and evaluation pathways differ. The current data do not establish whether the TOO output reduces diagnostic burden or improves clinical outcomes.

### 4.5 Exploratory Stage Classification

Stage classification did not reach a level that supports clinical triage, given the small cohort and preliminary nature of this study. Although the early‐versus‐late AUC was 0.866, late‐stage sensitivity was 26% at the reported operating point, and the four‐class model had low sensitivity for Stages II–IV. Notably, Stage I cancers were highly represented in the study cohort (*N* = 174) compared to other stages (Stage II *N* = 83, Stage III *N* = 63, and Stage IV *N* = 35). This was designed to capture early cancer volatomic signals. Late‐stage and metastatic cancers may express heterogeneous metabolic activity, which would reduce the stage classification sensitivity. Overall accuracy was inflated by the larger early‐stage group. Future work should prespecify the clinically relevant positive class, use balanced multisite cohorts, report calibration and class‐specific uncertainty, and validate the model independently.

### 4.6 Limitations and Future Directions

This study was retrospective and case–control. Pipeline development and performance estimation occurred within the same overall cohort, and no independent holdout or external validation cohort was evaluated. The case‐enriched design cannot estimate positive predictive value, negative predictive value, diagnostic yield, interval‐cancer rate, harms, or clinical utility in an asymptomatic population. Stage and grade were unavailable for some cases, and not all cancer types were represented at every stage.

One participating site contributed all controls and 59 cancer cases. The other two sites contributed cancer cases only. This imbalance could allow site‐associated signals to recur across participant‐level folds. The estimate from the site‐restricted case–control analysis was lower than the pooled estimate. The study cohort differed by age, sex, and race and did not represent a screening population. These imbalances in study cohort representation would be addressed with an appropriate screening population in a follow‐up study.

Tissue and stage outputs occasionally produced anatomically implausible or clinically weak classifications. Post hoc sex‐based constraints could reduce some impossible labels, but such rules must be prespecified and validated and do not remedy underlying model or cohort bias.

Pre‐analytical and clinical covariates were incompletely characterized. Collection time, collection‐to‐cooling interval, collection‐to‐freezing interval, fasting status, previous freeze–thaw history, smoking, diet, medication use, renal function, infection, comorbidity, recent procedures, and hospitalization were unavailable. These variables may affect urinary volatomic profiles and require prospective control or measurement.

## 5 Conclusions

Urinary VOC profiles distinguish known cancer cases from cancer‐free controls under internal cross‐validation. The feature‐removal analysis supported a distributed composite signal across multiple VOCs. Grouped TOO classification was promising, while individual tissue and stage results remained exploratory. The retrospective case–control design and absence of independent validation prevent conclusions about population‐screening accuracy or clinical utility. The next study should use a prespecified locked protocol, blinded analysis, and independent multicentric recruitment of cancer cases and controls from the intended‐use population. Reporting should follow current AI prediction model and diagnostic‐accuracy guidance [54, 55].

## Author Contributions

**Scott Campit:** methodology, software, validation, formal analysis, investigation, writing – original draft, writing – review and editing, visualization. **Sudharshanan Balaji:** validation, writing – review and editing. **Matthew Laskowski:** conceptualization, methodology, validation, formal analysis, resources, writing – review and editing, writing – original draft, project administration, supervision, funding acquisition, investigation, data curation. **Thomas J. Polascik:** validation, writing – review and editing.

## Funding

This work was supported by Toby Inc.

## Disclosure

Confidential patent applications related to the TOBY MCED Test have been filed.

## Ethics Statement

This study analyzed de‐identified urine specimens and associated clinical data obtained from three specimen providers; the authors' institution did not recruit participants or collect specimens. BioIVT specimens were collected under a protocol approved by WCG IRB (protocol 2010‐017; IRB tracking number 20161665; “Prospective Collection of Biological Specimens from Subjects Presenting at Specimen Donation Centers for Research”), with continuing review approved August 9, 2024. Specimens supplied through iSpecimen were collected under approvals issued by the research ethics committees of the contributing collection sites: for Site 116, a national ethics committee operating under ICH‐GCP and Ukrainian law (Ethical Review Numbers 2003‐20, 2003‐21, and 2003‐23, covering November 2020–March 2024); and for Site 597, a Non‐Interventional Clinical Research Ethics Committee in Türkiye (application 56812/2024; decision 489‐11, May 9, 2024). Site identities are withheld under confidentiality agreements with the supplier. Eastern Virginia Medical School specimens were collected under the EVMS Biorepository protocol (IRB #15‐10‐FB‐0195), reviewed by the Macon & Joan Brock Virginia Health Sciences at Old Dominion University IRB (FWA 00003956; IRB registrations IRB00000460 and IRB00001345), with continuing review approved on January 20, 2026. Participants provided written informed consent for research use of their specimens under the applicable site protocol, except where the reviewing board granted a waiver of consent and of HIPAA authorization for banked specimens and associated data. The study was conducted in accordance with the Declaration of Helsinki.

## Consent

Informed consent was obtained from all subjects for the use of their urine specimens and de‐identified demographic and clinical data for research purposes.

## Conflicts of Interest

Scott Campit is an employee, and Matthew Laskowski is the Chief Executive Officer of TOBY Inc. The other authors declare no conflicts of interest.

## Supporting information


**Figure S1:** SHAP summary plot for the 20 highest ranked features in the cancer‐signal detection model.

**Figure S2:** Exploratory tissue‐of‐origin classification among cancer cases. (A) One‐versus‐rest receiver operating characteristic curves. (B) Feature‐importance summary. (C) Top‐1 confusion matrix. (D) Top‐2 confusion matrix.

**Table S1:** Participant demographics, clinical characteristics, and participating site.

**Table S2:** Known and candidate biomarkers detected by TOBY models.

## Data Availability

The data that support the findings of this study are available from the corresponding author upon reasonable request.
