# Supplementary material for: Artificial Intelligence‐Based Urine Volatomic Assay for Multi‐Cancer Detection: A Multicentric Case–Control Discovery and Internal Validation Study
Source: Cancer Rep (Hoboken). 2026 Nov 10;9(11):e70735. doi: 10.1002/cnr2.70735 (PMC13749275; doi:10.1002/cnr2.70735)
Supplement: Supplementary file 1 [file CNR2-9-e70735-s003.docx]

**Supplementary Table 2.** Known and candidate biomarkers detected by TOBY models.

| **ID** | **Is Urine-Specific VOC?** | **Is Cancer Biomarker?** | **Cancer Type** | **PMID(s)** | **Log2 FC** | **U-Statistic** | **p-value** | **q-value** |
| --- | --- | --- | --- | --- | --- | --- | --- | --- |
| TOBY-VOC-001 | FALSE | TRUE | Lung (breath); Oral (saliva); Prostate (cell-line exometabolome) | 28440225; 33387033; 29615722 | -0.6291 | 11709 | 3.25E-16 | 1.88E-15 |
| Hydroxyacetone | TRUE | FALSE | Pancreatic (PDAC) | 33128797 | -0.7218 | 9421.5 | 4.73E-19 | 4.92E-18 |
| TOBY-VOC-003 | FALSE | TRUE | Melanoma | 24039618 | 1.0077 | 51726.5 | 2.28E-33 | 5.94E-32 |
| Decanal | TRUE | FALSE | Renal cell carcinoma | 27347408 | 0.0936 | 15527 | 6.11E-01 | 6.75E-01 |
| Stearic Acid | TRUE | FALSE | Ovarian carcinoma vs. benign tumor | 35503418 | 0.6142 | 14621.5 | 1.58E-06 | 5.14E-06 |
| TOBY-VOC-006 | FALSE | FALSE |  |  | -0.8955 | 1955 | 1.12E-04 | 2.78E-04 |
| TOBY-VOC-008 | FALSE | TRUE | Melanoma | 24039618 | -0.4496 | 6438 | 2.44E-01 | 3.17E-01 |
| TOBY-VOC-009 | FALSE | FALSE | Lung cancer (plasma lipidomics only) |  |  |  |  |  |
| TOBY-VOC-010 | FALSE | FALSE |  |  |  |  |  |  |
| TOBY-VOC-012 | FALSE | FALSE |  |  | -1.5578 | 417 | 4.56E-07 | 1.58E-06 |
| Gamma-Dodecalactone | TRUE | FALSE | Bladder cancer | 35956972 |  |  |  |  |
| Nonanal | TRUE | FALSE | Renal cell carcinoma; Bladder cancer; Pancreatic cancer (+ well-replicated lung-cancer breath evidence) | 27347408; 40389471; 33076134 | 0.0803 | 34160.5 | 3.40E-02 | 5.70E-02 |
| TOBY-VOC-015 | FALSE | FALSE |  |  |  |  |  |  |
| Decanoic Acid | TRUE | FALSE | Colorectal adenoma (Lynch-syndrome surveillance) | 40985860 |  |  |  |  |
| TOBY-VOC-017 | FALSE | FALSE |  |  | -0.4606 | 14704 | 2.22E-04 | 5.25E-04 |
| Acetic Acid | TRUE | FALSE | Gastro-esophageal cancer | 23421902 | -0.2381 | 15062 | 1.66E-01 | 2.33E-01 |
| TOBY-VOC-019 | FALSE | FALSE |  |  |  |  |  |  |
| TOBY-VOC-023 | FALSE | FALSE |  |  | 0.2746 | 5142 | 1.55E-01 | 2.24E-01 |
| Tetradecanoic acid | TRUE | FALSE | Prostate cancer | 38335180 | -0.0745 | 6443.5 | 1.74E-01 | 2.38E-01 |
| TOBY-VOC-026 | FALSE | FALSE |  |  |  |  |  |  |
| gamma-Butyrolactone | TRUE | FALSE | Bladder cancer | 35956972 | -0.9092 | 5032 | 6.14E-28 | 1.06E-26 |
| Heptanal | TRUE | FALSE | Lung cancer | 21763805 |  |  |  |  |
| TOBY-VOC-029 | FALSE | TRUE | Lung cancer (NSCLC) | 34083687 |  |  |  |  |
| TOBY-VOC-031 | FALSE | FALSE |  |  |  |  |  |  |
| phenol | TRUE | FALSE | Gastrointestinal and prostate cancer | 33383923 | 0.0591 | 7826.5 | 9.26E-01 | 9.62E-01 |
| TOBY-VOC-036 | FALSE | TRUE | Lung cancer | 36449484 |  |  |  |  |
| TOBY-VOC-038 | FALSE | FALSE |  |  |  |  |  |  |
| 4-Heptanone | TRUE | FALSE | Prostate cancer; Renal cell carcinoma | 27347408 |  |  |  |  |
| TOBY-VOC-040 | FALSE | FALSE |  |  |  |  |  |  |
| TOBY-VOC-041 | FALSE | FALSE |  |  |  |  |  |  |
| P-CRESOL | TRUE | FALSE | Gastric cancer; Leukemia/lymphoma/colorectal cancer | 27589838 | -0.1210 | 8063.5 | 4.98E-01 | 5.75E-01 |
| TOBY-VOC-046 | FALSE | FALSE |  |  |  |  |  |  |
| TOBY-VOC-047 | FALSE | TRUE | Bladder cancer | 30830384 | 0.3938 | 10818 | 8.35E-05 | 2.17E-04 |
| TOBY-VOC-048 | FALSE | TRUE | Colorectal cancer | 31877489 | -1.0262 | 2064 | 5.15E-09 | 2.43E-08 |
| TOBY-VOC-050 | FALSE | FALSE |  |  | -0.4178 | 2470 | 2.63E-01 | 3.33E-01 |
| TOBY-VOC-051 | FALSE | TRUE | Breast cancer (early-stage) | 35453141 |  |  |  |  |
| TOBY-VOC-053 | FALSE | FALSE |  |  |  |  |  |  |
| TOBY-VOC-054 | FALSE | FALSE |  |  | -0.6602 | 872 | 3.29E-04 | 7.37E-04 |
| TOBY-VOC-056 | FALSE | FALSE |  |  |  |  |  |  |
| TOBY-VOC-057 | FALSE | FALSE |  |  |  |  |  |  |
| TOBY-VOC-062 | FALSE | TRUE | Colorectal cancer / colon adenoma | 26721667; 33913149 |  |  |  |  |
| TOBY-VOC-065 | FALSE | FALSE |  |  |  |  |  |  |
| TOBY-VOC-066 | FALSE | TRUE | Non-small-cell lung cancer | 25634546 |  |  |  |  |
| TOBY-VOC-067 | FALSE | FALSE |  |  |  |  |  |  |
| TOBY-VOC-070 | FALSE | FALSE |  |  |  |  |  |  |
| TOBY-VOC-072 | FALSE | FALSE |  |  | -0.2234 | 12462 | 2.99E-01 | 3.61E-01 |
| TOBY-VOC-075 | FALSE | FALSE |  |  |  |  |  |  |
| TOBY-VOC-076 | FALSE | FALSE |  |  |  |  |  |  |
| 1-Undecanol | TRUE | FALSE | Colorectal cancer | 34450881 | 2.1274 | 43446 | 8.52E-41 | 4.43E-39 |
| Methanol | TRUE | FALSE | Gastro-oesophageal adenocarcinoma | 23421902 | -1.4963 | 13708 | 3.27E-18 | 2.43E-17 |
| 1-Hexadecanol | TRUE | FALSE | Prostate cancer (recurrence after radical prostatectomy); hepatocellular carcinoma (saliva) | 40940853; 37760495 |  |  |  |  |
| 4-Isopropylbenzaldehyde | TRUE | FALSE | Lung cancer; colorectal cancer / lymphoma (occurrence only) | 35050157; 22085842 |  |  |  |  |
| TOBY-VOC-081 | FALSE | TRUE | Gastric cancer (MGC-803 cells vs GES-1 normal gastric mucosa cells) | 24465273 | 0.3374 | 10588 | 5.30E-02 | 8.62E-02 |
| TOBY-VOC-082 | FALSE | TRUE | Prostate cancer (DU145 cell line vs PNT2) | 29615722 |  |  |  |  |
| TOBY-VOC-083 | FALSE | TRUE | Colorectal cancer; lung cancer | 38338911; 22071773; 42253921 |  |  |  |  |
| TOBY-VOC-084 | FALSE | FALSE |  |  | 0.8678 | 39101.5 | 2.51E-17 | 1.63E-16 |
| TOBY-VOC-085 | FALSE | FALSE |  |  | -0.7894 | 15587 | 1.70E-19 | 2.21E-18 |
| TOBY-VOC-086 | FALSE | FALSE |  |  |  |  |  |  |
| TOBY-VOC-087 | FALSE | FALSE |  |  |  |  |  |  |
| TOBY-VOC-088 | FALSE | TRUE | Lung adenocarcinoma (A549 cell line vs normal lung cell lines) | 22364569 |  |  |  |  |
| Estradiol | TRUE | FALSE | Breast (premenopausal; postmenopausal) | 22144471; 27864342 | -0.3079 | 2435 | 4.60E-04 | 9.58E-04 |
| Estrone | TRUE | FALSE | Breast (premenopausal) | 22144471 | -0.5351 | 18015 | 5.39E-08 | 2.16E-07 |
| Prasterone Sulfate (DHEA-S) | TRUE | FALSE | Prostate (vs BPH, urinary extracellular vesicles); breast (tentative); bladder (serum, pre-diagnostic) | 29760869; 42271577; 1825478 | -0.5351 | 18015 | 5.39E-08 | 2.16E-07 |
| Valeric Acid | TRUE | FALSE | Renal cell carcinoma | 26018279 | 0.3147 | 2209 | 2.93E-01 | 3.61E-01 |
| Theaspirane | TRUE | FALSE | Prostate cancer | 31588123; 41375043 | -1.6692 | 2225 | 2.40E-07 | 8.91E-07 |
| TOBY-VOC-094 | FALSE | TRUE | Colorectal cancer (vs colorectal adenoma) | 36174955 |  |  |  |  |
| TOBY-VOC-095 | FALSE | TRUE | Breast cancer | 16502014; 32992445 |  |  |  |  |
| TOBY-VOC-096 | FALSE | TRUE | Colorectal cancer (breath); hepatocellular carcinoma (saliva) | 38338911; 34541549 | -0.2067 | 26232 | 2.38E-03 | 4.77E-03 |
| TOBY-VOC-097 | FALSE | TRUE | Mixed cancers (carcinoma, lymphoma, leukaemia) | 31409861 |  |  |  |  |
| TOBY-VOC-098 | FALSE | FALSE |  |  |  |  |  |  |
| TOBY-VOC-099 | FALSE | FALSE |  |  | -0.9643 | 1580 | 1.99E-10 | 1.03E-09 |

Undisclosed compounds are shown by their masked TOBY-VOC identifier. Log2 FC, U-statistic, p and q (Benjamini-Hochberg) are the run's cancer-versus-control differential-abundance statistics; blank = not tested.
