# Supplementary material for: Artificial Intelligence‐Based Urine Volatomic Assay for Multi‐Cancer Detection: A Multicentric Case–Control Discovery and Internal Validation Study
Source: Cancer Rep (Hoboken). 2026 Nov 10;9(11):e70735. doi: 10.1002/cnr2.70735 (PMC13749275; doi:10.1002/cnr2.70735)
Supplement: Supplementary file 2 [file CNR2-9-e70735-s004.docx]

**Supplementary Table 1.** Participant demographics, clinical characteristics, and participating site.

| **Characteristic** | **BioIVT (n=59)** | **EVMS (n=101)** | **iSpecimen (n=331)** | **All cancers (n=491)** |
| --- | --- | --- | --- | --- |
| Age, years ± SD (range) | 66.2 ± 8.7 (44-80) | 63.2 ± 10.1 (39-88) | 56.4 ± 8.6 (29-75) | 59.0 ± 9.7 (29-88) |
| **Sex, n (%)** |  |  |  |  |
| Male | 44 (74.6%) | 73 (72.3%) | 77 (23.3%) | 194 (39.5%) |
| Female | 15 (25.4%) | 28 (27.7%) | 254 (76.7%) | 297 (60.5%) |
| **Race, n (%)** |  |  |  |  |
| Caucasian | 59 (100.0%) | 72 (71.3%) | 331 (100.0%) | 462 (94.1%) |
| African American | 0 (0.0%) | 21 (20.8%) | 0 (0.0%) | 21 (4.3%) |
| Hispanic/Latino | 0 (0.0%) | 0 (0.0%) | 0 (0.0%) | 0 (0.0%) |
| Pacific Islander | 0 (0.0%) | 2 (2.0%) | 0 (0.0%) | 2 (0.4%) |
| Asian | 0 (0.0%) | 0 (0.0%) | 0 (0.0%) | 0 (0.0%) |
| Unknown | 0 (0.0%) | 6 (5.9%) | 0 (0.0%) | 6 (1.2%) |
| **Cancer Type, n (%)** |  |  |  |  |
| Breast | 0 (0.0%) | 0 (0.0%) | 84 (25.4%) | 84 (17.1%) |
| Prostate | 0 (0.0%) | 24 (23.8%) | 0 (0.0%) | 24 (4.9%) |
| Lung | 0 (0.0%) | 0 (0.0%) | 25 (7.6%) | 25 (5.1%) |
| Colorectal | 0 (0.0%) | 0 (0.0%) | 50 (15.1%) | 50 (10.2%) |
| Bladder | 58 (98.3%) | 29 (28.7%) | 0 (0.0%) | 87 (17.7%) |
| Kidney | 1 (1.7%) | 48 (47.5%) | 0 (0.0%) | 49 (10.0%) |
| Pancreas | 0 (0.0%) | 0 (0.0%) | 25 (7.6%) | 25 (5.1%) |
| Stomach | 0 (0.0%) | 0 (0.0%) | 50 (15.1%) | 50 (10.2%) |
| Ovarian | 0 (0.0%) | 0 (0.0%) | 65 (19.6%) | 65 (13.2%) |
| Cervical | 0 (0.0%) | 0 (0.0%) | 32 (9.7%) | 32 (6.5%) |
| **Tumor Grade, n (%)** |  |  |  |  |
| 1 | 0 (0.0%) | 0 (0.0%) | 17 (5.1%) | 17 (3.5%) |
| 2 | 0 (0.0%) | 0 (0.0%) | 51 (15.4%) | 51 (10.4%) |
| 3 | 0 (0.0%) | 0 (0.0%) | 16 (4.8%) | 16 (3.3%) |
| 4 | 0 (0.0%) | 0 (0.0%) | 0 (0.0%) | 0 (0.0%) |
| Unknown | 59 (100.0%) | 101 (100.0%) | 247 (74.6%) | 407 (82.9%) |
| **AJCC Stage Group, n (%)** |  |  |  |  |
| I | 0 (0.0%) | 0 (0.0%) | 174 (52.6%) | 174 (35.4%) |
| II | 9 (15.3%) | 0 (0.0%) | 74 (22.4%) | 83 (16.9%) |
| III | 10 (16.9%) | 0 (0.0%) | 53 (16.0%) | 63 (12.8%) |
| IV | 3 (5.1%) | 2 (2.0%) | 30 (9.1%) | 35 (7.1%) |
| Unknown | 37 (62.7%) | 99 (98.0%) | 0 (0.0%) | 136 (27.7%) |
| **Stage Resolution, n (%)** |  |  |  |  |
| Exact AJCC group (I-IV) | 22 (37.3%) | 2 (2.0%) | 331 (100.0%) | 355 (72.3%) |
| Bounded by TNM to III/IV | 1 (1.7%) | 18 (17.8%) | 0 (0.0%) | 19 (3.9%) |
| Bounded by TNM to 0/I/II | 0 (0.0%) | 0 (0.0%) | 0 (0.0%) | 0 (0.0%) |
| Unresolved | 36 (61.0%) | 81 (80.2%) | 0 (0.0%) | 117 (23.8%) |
| **Gleason Score, n (%)** |  |  |  |  |
| 6 | 0 (0.0%) | 12 (11.9%) | 0 (0.0%) | 12 (2.4%) |
| 7 | 0 (0.0%) | 11 (10.9%) | 0 (0.0%) | 11 (2.2%) |
| 8 | 0 (0.0%) | 1 (1.0%) | 0 (0.0%) | 1 (0.2%) |
| 9-10 | 0 (0.0%) | 0 (0.0%) | 0 (0.0%) | 0 (0.0%) |
| Not applicable (non-prostate) | 59 (100.0%) | 77 (76.2%) | 331 (100.0%) | 467 (95.1%) |
| Unknown | 0 (0.0%) | 0 (0.0%) | 0 (0.0%) | 0 (0.0%) |
| **Risk Group, n (%)** |  |  |  |  |
| Low-Risk | 0 (0.0%) | 12 (11.9%) | 0 (0.0%) | 12 (2.4%) |
| High-Risk | 0 (0.0%) | 12 (11.9%) | 0 (0.0%) | 12 (2.4%) |
| Not applicable (non-prostate) | 59 (100.0%) | 77 (76.2%) | 331 (100.0%) | 467 (95.1%) |
| Unknown | 0 (0.0%) | 0 (0.0%) | 0 (0.0%) | 0 (0.0%) |
